# Adrenal Metastasis of Hepatocellular Carcinoma With Unknown Primary: A Case Report

**DOI:** 10.7759/cureus.102663

**Published:** 2026-01-30

**Authors:** Colleen Conger, John Yassa, Mohit Agarwal, Sukeshi Arora, Neil Newman

**Affiliations:** 1 Radiation Oncology, AdventHealth Redmond, Rome, USA; 2 Radiation Oncology, University of Texas Health Science Center at San Antonio, San Antonio, USA; 3 Radiation Oncology, University of Texas Health San Antonio MD Anderson Cancer Center, San Antonio, USA; 4 Radiation Oncology, CommonSpirit Health, Pueblo, USA; 5 Hematology and Oncology, University of Texas Health Science Center at San Antonio, San Antonio, USA

**Keywords:** adrenal metastases, hepatocellular carcinoma (hcc), metastatic disease of unknown primary, radiation oncoolgy, sbrt (stereotactic body radiotherapy)

## Abstract

Solitary extrahepatic hepatocellular carcinoma (HCC) with an unknown primary has been rarely reported in the literature. We report a case of a 69-year-old Hispanic male with no history of malignancy presenting with a solitary 5cm adrenal mass found incidentally on CT surveillance for high-risk liver disease secondary to chronic hepatitis C (HCV). Biopsy returned as metastatic poorly differentiated hepatocellular carcinoma. A dedicated liver MRI was performed to evaluate for a primary site, but no dominant lesion was noted. Follow-up abdominal CT two months later revealed interval enlargement of the mass to 6.2cm with an alpha-fetoprotein (AFP) of 169 ng/ml. After a multidisciplinary discussion, this was deemed a tumor of unknown primary.

The patient was offered definitive treatment with stereotactic body radiotherapy (SBRT) 60 Gy in 8 fractions, with careful attention paid to the stomach, resulting in undercoverage of the lesion. AFP levels were used to track treatment efficacy and disease response. Retesting of AFP three months later was 20 ng/ml. CT imaging at the three-month follow-up was also consistent with a marked decrease to 3.6 cm from 6.2 cm. 27 months after treatment completion, the patient remains without signs of disease progression and normal AFP of 14.4 ng/ml without any clinical or radiographic signs of recurrence.

HCC with solitary extrahepatic presentation is rare. Treatment with SBRT alone, and no systemic therapy, to an extrahepatic metastasis can yield excellent local and biochemical control, as expected based on the literature for primary SBRT to HCC lesions. This case underscores the importance of cautious attention to sparing of normal organs at risk, such as the stomach, since HCC tumors can respond to subablative doses. High-dose SBRT can be used to definitively treat oligometastatic hepatocellular carcinoma with an unknown primary tumor; however, longer-term follow-up will be needed to assess local control and whether new metastases arise.

## Introduction

As of 2018, primary liver cancer is the seventh most common cancer worldwide, with 75% of new cases being hepatocellular carcinoma (HCC) [1]. In 2019, 35,563 new cases of liver cancer and 27,958 deaths due to liver cancer were reported in the United States alone [2], with an estimated 80% of these being HCC [3]. Risk factors for HCC include American Indian/ Alaska native race [4], HBV and HCV infection [5], excessive alcohol consumption [6], metabolic syndrome, including diabetes and obesity [7], and NAFLD. 

Extrahepatic metastases of HCC are most common in the lungs, abdominal lymph nodes, and bones [8]. Metastases to the adrenal glands occur in about 10% of cases [9]. Metastasis almost exclusively occurs when the primary tumor is stage III or IVA [9]. Despite the frequency of HCC metastasis, HCC rarely presents as a metastatic tumor of unknown primary. Only isolated cases have been reported, with the prevalence of HCC of unknown primary remaining unknown [10-15].

The currently agreed-upon first-line therapy for advanced unresectable or metastatic HCC is immunotherapy [16-17]. Stereotactic body radiotherapy (SBRT) has also previously demonstrated excellent local control of advanced primary liver disease (95% at 5 years [18], with an overall survival benefit in a phase 3 randomized trial showing no increased rates of grade 3 toxicity [19]. We present a case in which SBRT was used to treat HCC of unknown primary, with the only known site of disease in the adrenal glands.

## Case presentation

**Figure 1:**
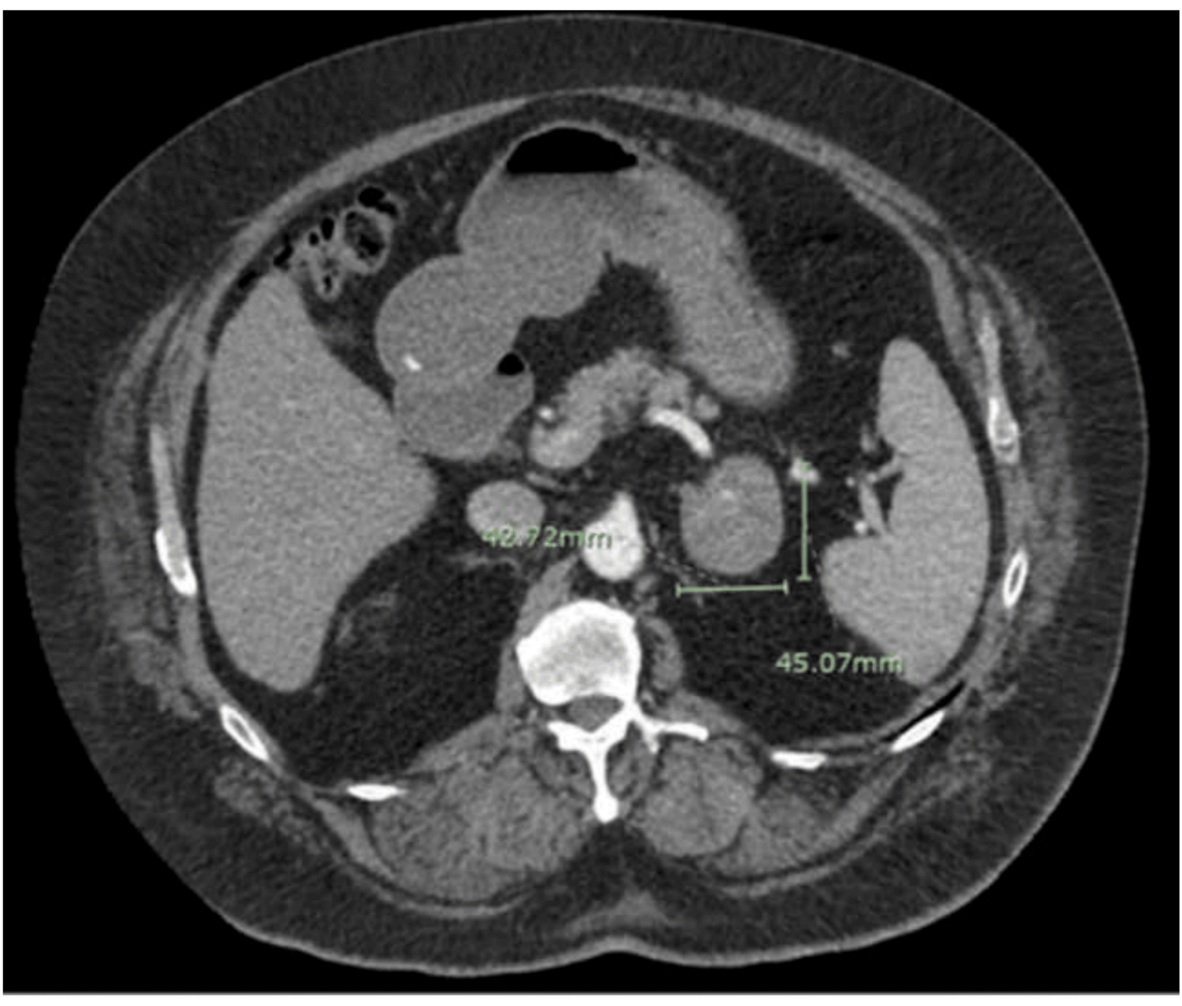
Abdominal CT 3-phase demonstrating a 4.2 x 4.5 cm adrenal mass.

A 69-year-old Hispanic male, with a history of decompensated liver disease secondary to hepatitis C (HCV) cirrhosis (Child-Pugh class A6) treated with Harvoni (ledipasvir/sofosbuvir) with sustained viral response, presented due to an oligometastatic adrenal mass of unknown origin. The solitary 4.2 x 4.5 cm indeterminate adrenal mass was found incidentally while he was receiving CT surveillance for his high-risk liver disease (Figure 1). The mass was concerning for malignancy, as comparison CT imaging from eight months prior did not reveal an adrenal mass. At the time of diagnosis, the liver was noteworthy for only mild nodular liver margins with a slightly enlarged caudate lobe, but no masses on CT. The patient’s alpha-fetoprotein (AFP) level at this time was elevated to 27.6 ng/ml (normal = <20). Biopsy of the mass returned as metastatic poorly differentiated HCC.

**Figure 2:**
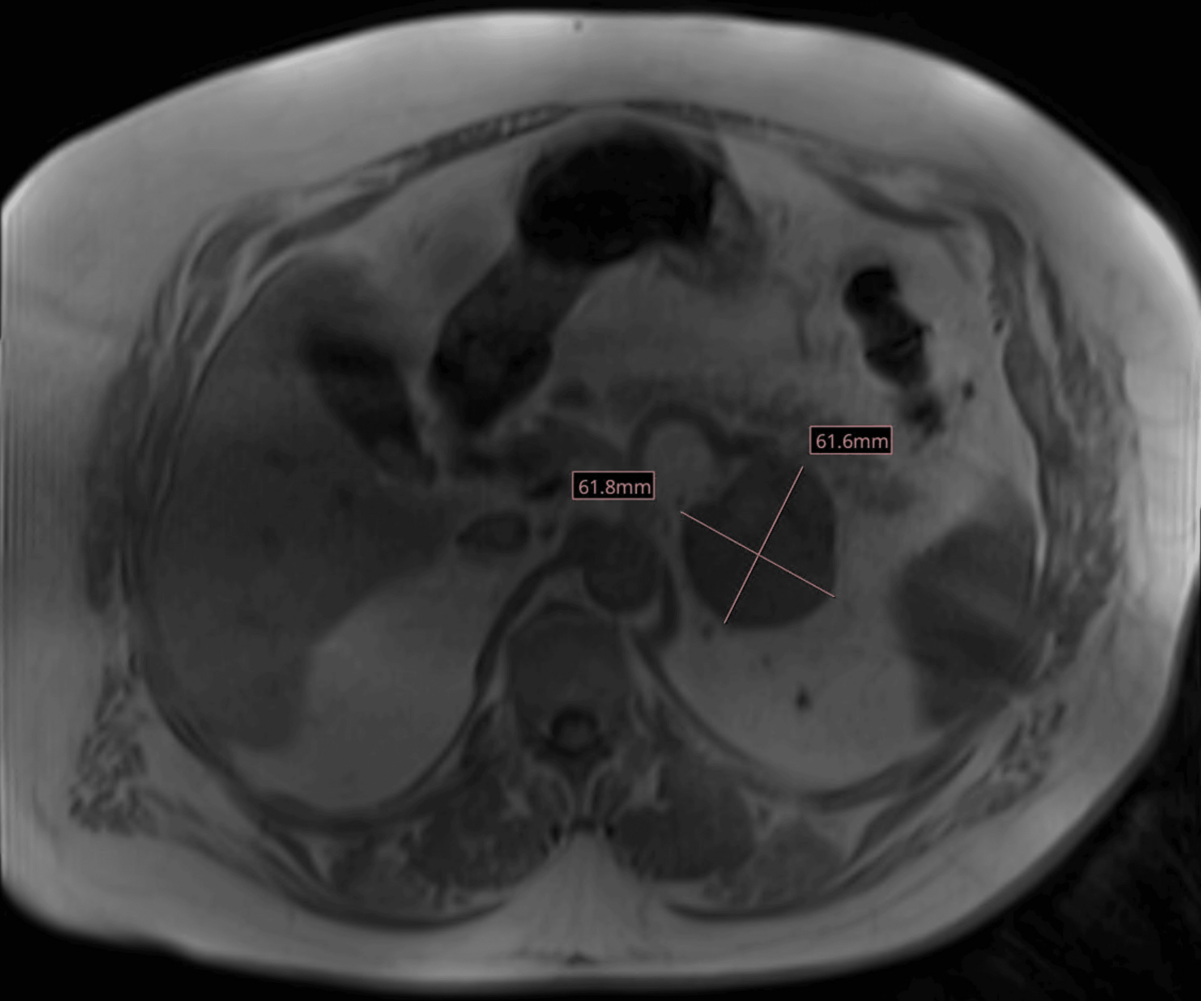
Abdominal MRI showing interval enlargement of the mass to 6.2 x 6.2 cm.

A dedicated liver MRI was performed to evaluate for a possible HCC primary site, but no dominant lesion was noted. Follow-up abdominal MRI two months later revealed interval enlargement of the mass to 6.2 x 6.2 cm (Figure 2). It was noted to be heterogeneously enhancing and centrally necrotic, with a moderate thrombus in the left adrenal vein. Further lab workup at this time revealed an AFP of 118.6 ng/ml. After a multidisciplinary discussion, this was deemed a tumor of unknown primary.

The patient was offered definitive treatment with SBRT to 60 Gy in 8 fractions, with careful attention paid to the stomach, which bordered the superior 2 cm of the tumor. The patient was simulated supine, arms up, in a Vac-Lok bag, with IV contrast, after fasting for 6 hours prior to simulation. He had a 4-dimensional CT scan, with which the tumor motion was tracked in space throughout his entire respiratory cycle. 

**Figure 3:**
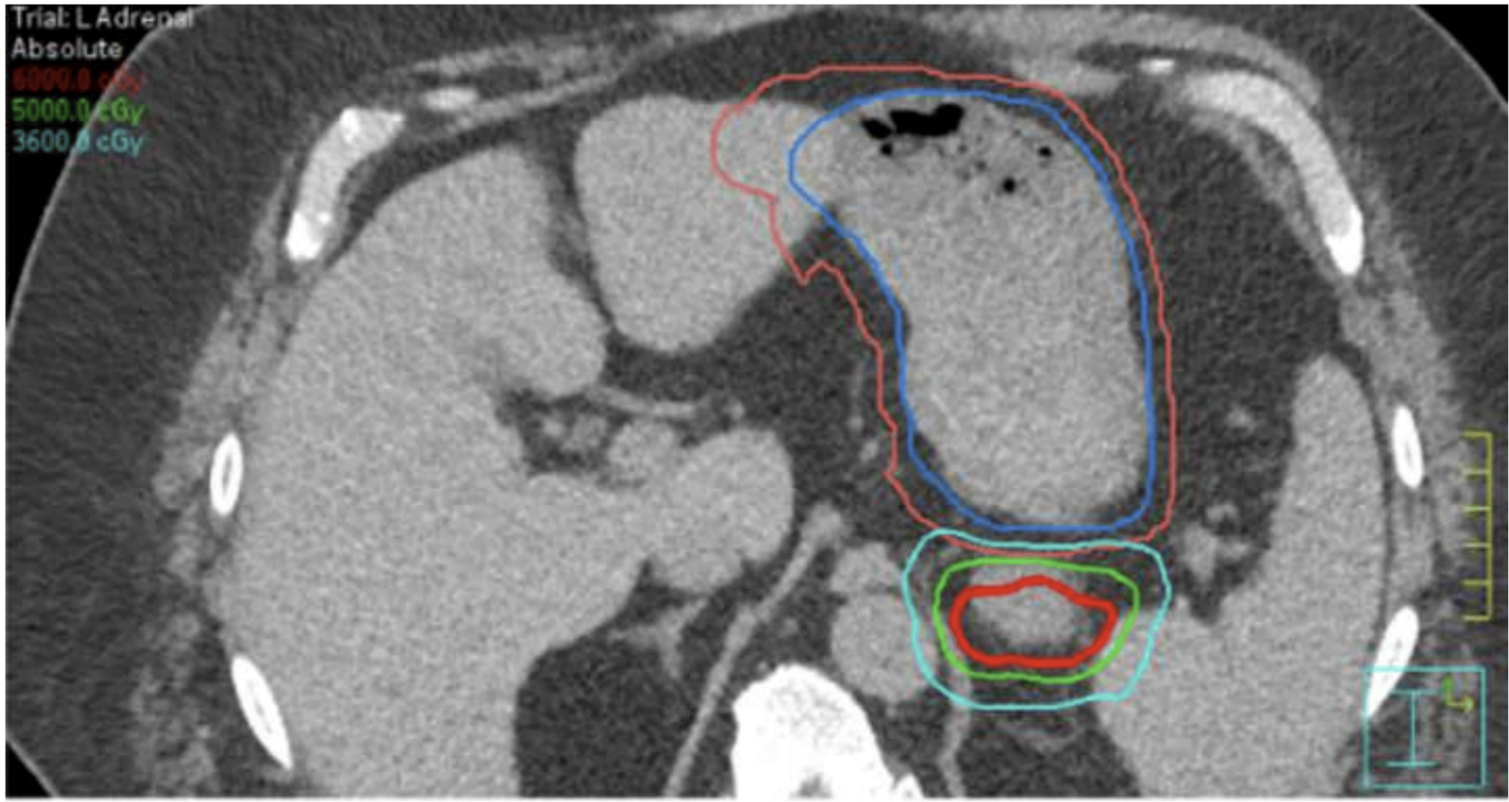
Isodose lines of the tumor and stomach. Note the proximity of the stomach to the superior 2 cm of the tumor.

The gross tumor volume (GTV) was 128 cc, with a planned target volume (PTV) expansion to 195 cc (Figure 3). The stomach was constrained to a dose of 5 cc (D5cc) of less than 31 Gy and a D0.035cc of less than 40 Gy. A planning organ at risk volume (PRV) was created for the stomach and small intestines and kept to a 40 Gy maximum. These PRVs were comprised of the motion of these structures in the 4D CT with an additional 0.5cm margin. Daily imaging required cone beam CT scans, with isodose lines imported into the structures.

**Figure 4:**
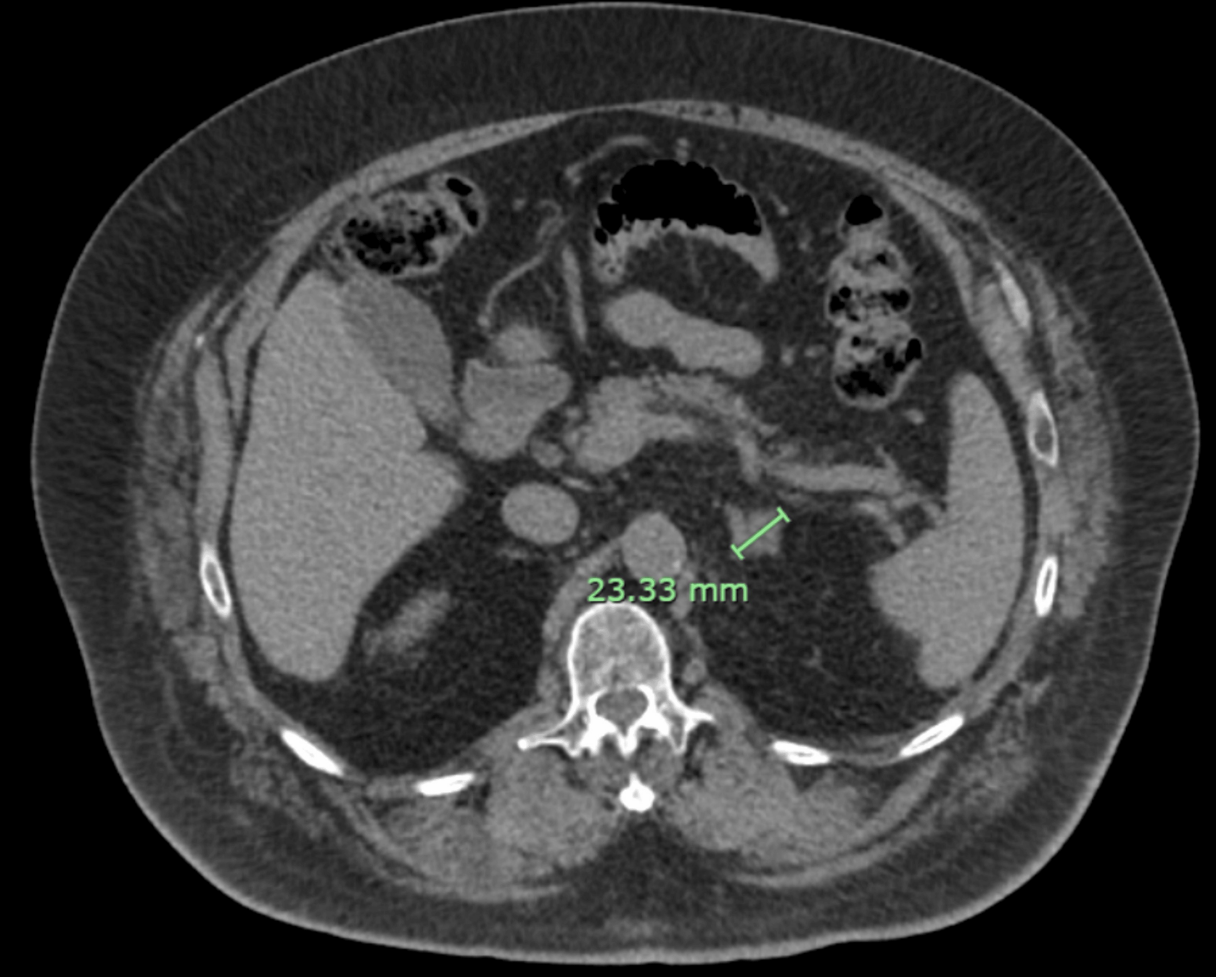
One-year follow-up abdominal CT 3-Phase showing an interval decrease in tumor size to 2.3 cm

AFP levels as well as serial imaging were used to track treatment efficacy and disease response (Table 1). Two weeks after completion of treatment, the patient’s AFP was elevated (an expected acute reaction) to 169 ng/ml. Retesting three months later, however, showed a reduced AFP of 20 ng/ml. CT imaging at the one-year follow-up was also consistent with a marked decrease in the size of the tumor from 6.2 cm to 2.3 cm (Figure 4). At 12-month follow-up, his tumor had shrunk to 2.1 cm in greatest dimension, and his AFP was 19 ng/ml. At the time of this case report, 45 months after treatment completion, the patient remains without signs of disease progression. His most recent AFP was 8.16 ng/ml, and the mass size on imaging was 1.5 cm. This patient has not received any systemic therapy.

**Table 1:** Interval AFP values AFP: Alpha-fetoprotein

AFP Reference range	Pre-treatment AFP	Immediate post-treatment AFP	3 months post-treatment AFP	45 months post-treatment AFP
<20.0 ng/mL	27.6	169	20	8.16

## Discussion

HCC is the most common primary hepatic malignancy, particularly in patients with preexisting liver disease. The adrenal gland is the sixth most common site of metastasis after the lungs, peritoneum, bone, lymph nodes, and spleen [20]. This patient’s presentation of metastasis to the adrenal gland, with an unknown primary hepatic tumor, has not been previously reported. Previous literature on HCC metastases with unknown primary is currently limited to the cervical spine, pelvic bones, chest wall, skull, and sternum [10-15].

As of 2018, the current standard of treatment in the United States for HCC is systemic therapy utilizing immunotherapy with Atezolizumab and Bevacizumab or Durvalumab and Tremelimumab [17]. The use of radiotherapy in the treatment of HCC is currently only strongly recommended in the case of liver-confined multifocal or unresectable HCC [21]. Due to its rarity, there is no known literature on primary radiotherapy treatment alone of hepatocellular carcinoma metastases with an unknown primary tumor. 

**Figure 5:**
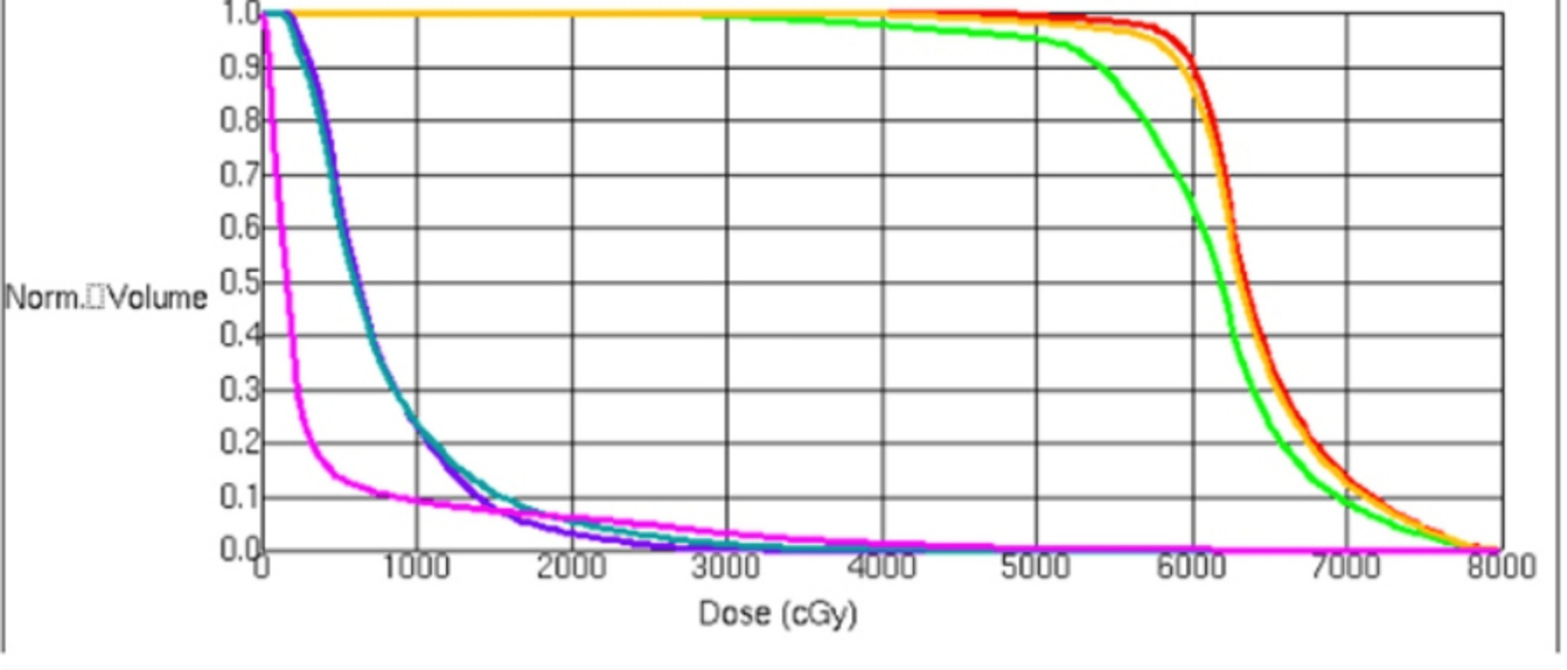
Dose volume histogram of left adrenal gland treatment plan. Color glossary: Red- GTV; Orange- ITV; Green- PTV; Purple- stomach; Teal- Stomach PRV; Pink- kidneys.

The current recommendation for treating liver-confined HCC with RT suggests a minimum BED10 of 6500 to 7900 cGy [22]. In series with large median tumor sizes, local control appears to be excellent with dose escalation18. However, despite this, HCC appears to be somewhat radiosensitive to high doses per fraction. Even doses as low as 30 Gy in 6 fractions (45 BED) or 35 Gy in 5 fractions [19] appear to have adequate local control [23]. This patient’s 6.2 x 6.2cm mass received a BED10 of 105 to the majority of the GTV. This was necessary to administer an adequate dose to the tumor, as the PTV could not include the whole tumor in areas that bordered the stomach. With this method, 95% of the GTV received a dose of 60 Gy, and 95% of the PTV received a dose of 50 Gy (Figure 2). The dose-volume histogram (DVH) demonstrates that the constraints met for this patient were within the recommended doses (Figure 5). It is imperative, as a principle with oligometastatic disease, to place more weight on the organ at risk over the tumor coverage, especially with a pathology that can be responsive to a lower total biological effective dose (BED).

This patient also never received any systemic therapy. While this is the standard of care for advanced/metastatic liver disease, a trial of total consolidation may be able to delay the time to systemic therapy. Systemic therapy has not only financial burdens to underserved patients, but also high rates of grade 3 toxicity.

## Conclusions

HCC with solitary extrahepatic presentation is rare. Treatment with SBRT alone to an extrahepatic metastasis can yield excellent local and biochemical control, as expected based on the literature for primary SBRT to HCC lesions. This case underscores the importance of cautious attention to sparing of normal organs at risk, such as the stomach, since HCC tumors can respond to BED doses as low as 45 Gy. This contrasts with other tumors requiring considerably higher BED in the same region. High-dose SBRT has been used for this patient to definitively treat oligometastatic hepatocellular carcinoma with an unknown primary tumor, with a short-interval encouraging biochemical and radiological response. However, longer-term follow-up will be needed to assess local control, whether new metastases arise, and longer-term toxicity in this patient. Further study in a larger cohort of patients to investigate whether SBRT alone could be effectively used for local control and delays to initiating systemic therapy could prove beneficial.
